# Validation of the depression anxiety stress scales (DASS) 21 as a screening instrument for depression and anxiety in a rural community-based cohort of northern Vietnamese women

**DOI:** 10.1186/1471-244X-13-24

**Published:** 2013-01-12

**Authors:** Thach Duc Tran, Tuan Tran, Jane Fisher

**Affiliations:** 1 Research and Training Centre for Community Development, Hai Ba Trung District, Hanoi, Viet Nam; 2 Jean Hailes Research Unit, School of Public Health and Preventive Medicine, Monash University, Clayton, Australia; 3 Melbourne School of Population Health, Australia, Monash University, Parkville, Australia

**Keywords:** Screening, Depression, Anxiety, Validation, Women, Vietnam

## Abstract

**Background:**

Depression and anxiety are recognised increasingly as serious public health problems among women in low- and lower-middle income countries. The aim of this study was to validate the 21-item Depression Anxiety and Stress Scale (DASS21) for use in screening for these common mental disorders among rural women with young children in the North of Vietnam.

**Methods:**

The DASS-21 was translated from English to Vietnamese, culturally verified, back-translated and administered to women who also completed, separately, a psychiatrist-administered Structured Clinical Interview for DSM IV Axis 1 diagnoses of depressive and anxiety disorders. The sample was a community-based representative cohort of adult women with young children living in Ha Nam Province in northern Viet Nam. Cronbach’s alpha, Exploratory Factor Analyses (EFA) and Receiver Operating Characteristic (ROC) analyses were performed to identify the psychometric properties of the Depression, Anxiety, and Stress subscales and the overall scale.

**Results:**

Complete data were available for 221 women. The internal consistency (Cronbach’s alpha) of each sub-scale and the overall scale were high, ranging from 0.70 for the Stress subscale to 0.88 for the overall scale, but EFA indicated that the 21 items all loaded on one factor. Scores on each of the three sub-scales, and the combinations of two or three of them were able to detect the common mental disorders of depression and anxiety in women with a sensitivity of 79.1% and a specificity of 77.0% at the optimal cut off of >33. However, they did not distinguish between those experiencing only depression or only anxiety.

**Conclusions:**

The total score of the 21 items of the DASS21-Vietnamese validation appears to be comprehensible and sensitive to detecting common mental disorders in women with young children in primary health care in rural northern Vietnam and therefore might also be useful to screen for these conditions in other resource-constrained settings.

## Background

Depression and anxiety are the common non-psychotic mental disorders experienced most frequently by women living in resource-constrained low and lower-middle income countries [1,2]. Early detection of these problems in primary health care is essential to identifying women who might be offered targeted treatments and therefore to improving prognosis among them and reducing disability in the community [3]. As the competing health priorities of infectious illness and malnutrition are diminishing in these settings, awareness of mental health is growing. There is an increasing need for screening tools, which primary health care staff can use, to identify people experiencing common mental disorders in the community.

The relationship between depression and anxiety has been of longstanding interest to clinicians and researchers. Clark and Watson [4] proposed a tripartite model of anxiety and depression. This model suggests that both conditions share several symptoms of elevated negative effect (e.g. distress and irritability). Depression is characterised by low levels of positive effect including experiences of happiness, confidence, and enthusiasm, while physiological hyperarousal is unique to anxiety. Even though depression and anxiety are conceptually distinct, there is substantial overlap between the two states that makes them difficult to distinguish in screening.

The Depression Anxiety and Stress Scales (DASS, [5]) is a widely used screening tool to assess symptoms of depression, anxiety, and stress in community settings. This instrument comprises three sub-scales: (1) the Depression sub-scale which measures hopelessness, low self-esteem, and low positive affect; (2) the Anxiety scale which assesses autonomic arousal, musculo-skeletal symptoms, situational anxiety and subjective experience of anxious arousal; and (3) the Stress scale which assesses tension, agitation, and negative affect. There are two forms of the DASS, the full 42-item and the short 21-item versions. Both assess the same domains.

There is evidence of the validity of the DASS for the use in both clinical and community settings in English-speaking countries including Australia [6,7], the United States of America [8], Canada [9], and England [10]. This tool has also been translated and validated in other languages including Chinese [11], Malay [12], Italian [13], and Spanish [14]. Both English and non-English versions have high internal consistency (Cronbach’s alpha scores of > 0.7). Previous investigations in English-speaking and non-English-speaking countries have found significant correlations between DASS scores and other measures including the Beck Anxiety and Beck Depression Scales (correlation coefficients, r, ranged from 0.58 to 0.78, [6]), the Positive and Negative Affect Scale (r = 0.69, [10]), and Symptom Checklist-90-R (r ranged from 0.57 to 0.84, [14]). However, there has not yet been a validation study of the DASS21 against a clinical diagnostic test to propose and verify cut-off scores to detect common mental disorders.

There is only a small set of psychometric instruments to detect symptoms of common mental disorders that have been validated for use in Vietnam. Our team has established the psychometric properties of Zung’s Self-rated Anxiety Scale, the Edinburgh Postnatal Depression Scale, and General Health Questionnaire 12 items for screening for common mental disorders including depression and anxiety during pregnancy and in the postpartum year [15] and the Self Reporting Questionnaire-20 items to establish psychiatric caseness in adult women [16]. The Depression Anxiety and Stress Scale is a brief screening instrument, which is of particular value because it can assess multiple domains and is psychometrically sound in Anglophone and no-Anglophone settings, but its comprehensibility and psychometric properties in resource-constrained countries have not yet been established [8].

The aim of this study was to establish the comprehensibility and psychometric properties including internal reliability and criterion validity of the Depression, Anxiety and Stress Scale as a screening instrument for depression and / or anxiety in women with young children living in rural northern Vietnam.

## Methods

The study used the gold-standard design for scale validation of comparing the responses to the instrument with an independently administered diagnostic interview.

### Participants

Participants were women living in Ha Nam Province in rural northern Viet Nam who had participated in a prior study [17] which established the prevalence and determinants of perinatal common mental disorders among them and had agreed to be informed about subsequent follow up studies. Ha Nam is a rural province located approximately 50 km south of Hanoi, the national capital. Ha Nam has a population of 0.8 million inhabitants and, in 2011, an average annual per capita income of USD800. Most people earn income from rice farming and making handcrafts and some are employed in local industries. The original study recruited women in Ha Nam through a two-stage sampling procedure. First, six communes were selected randomly by an independent statistician from a list of all 108 rural communes in the province as study sites. Communes are the primary administrative subdivisions in Vietnam and each has a population of about 5,000 to 10,000 people in Ha Nam. In each study site, all women registered with the commune health centres in the previous month as being either at least 7 months pregnant or between the fourth and eighth week postpartum were eligible and invited to participate in the study. Finally, 234 perinatal women were recruited in the original study with a recruitment fraction of 92%. They constituted a representative sample of women with young children in this province. This validation study was initiated eighteen months after data collection for the prior study had been completed.

### Measures

The 21-item Depression Anxiety and Stress Scales was selected for this validation. Each of the three sub-scales: (DASS21-D), Anxiety (DASS21-A), and Stress (DASS21-S) has seven items. Each item comprises a statement and four short response options to reflect severity and scored from 0 (*Did not apply to me at all*) to 3 (*Applied to me very much, or most of the time*). In order to yield equivalent scores to the full DASS 42, the total score of each scale is multiplied by two [5] and ranges from 0 to 42. In the normative sample based on 1870 Australian females aged 17 to 79 years, means (standard deviation) were 6.14 (6.92) for the DASS21-D sub-scale; 4.80 (5.03) for the DASS21-A subscale and 10.29 (8.16) for the DASS21-S subscale [5]. The DASS sub-scale severity ratings suggested for Australia are shown in Table 1.

**Table 1:** DASS21 sub-scale severity ratings suggested for Australian

**Severity**	**DASS21-D**	**DASS21-A**	**DASS21-S**
Normal	0-9	0-7	0-14
Mild	10-13	8-9	15-18
Moderate	14-20	10-14	19-25
Severe	21-27	15-19	26-33
Extremely severe	28+	20+	34+

DASS21-D: Depression sub-scale of The Depression Anxiety and Stress Scales 21 items; DASS21-A: Anxiety sub-scale of The Depression Anxiety and Stress Scales 21 items; and DASS21-S: Stress sub-scale of The Depression Anxiety and Stress Scales 21 items.

*Source:*[5]*.*

It was validated against individual psychiatrist-administered Structured Clinical Interviews for DSM IV Axis 1 Diagnoses (SCID) modules for depression (mild, moderate, and severe Major Depression or Dysthymia) and anxiety (Generalised Anxiety Disorder and Panic Disorder) in this study [18].

### Procedure

The DASS21 was translated from English into Vietnamese, reviewed by a group of health professionals and research workers for appropriateness of language and cultural idioms and back-translated to English for verification. Participants completed the DASS21-V as an individual structured interview with a trained health researcher. In a separate process, a Vietnamese psychiatrist administered SCID interviews on the same day. All participants completed both interviews in private rooms at the commune health station on the on the same day. The psychiatrist was blinded to scores on the DASS21 and vice versa. Data collection was carried out in February 2008.

### Statistical analysis

Cronbach’s alpha coefficients were calculated to measure the internal reliability for each sub-scale with a cut-off of 0.7 used to indicate high internal reliability. Exploratory Factor Analyses utilizing Varimax rotation (EFAs) were performed to investigate the internal structure of all items and of each sub-scale. The criterion chosen to determine that an extracted factor accounted for a reasonably large proportion of the total variance was based on an eigenvalue greater than one [19]. The significance of an item factor loading was set at a coefficient level of 0.30 or greater [20].

Medians and interquartile ranges of DASS21 sub-scale scores and all items were calculated by diagnostic groups due to the skewed distributions. Nonparametric K-sample tests were performed to test the equality of medians. The Area Under the ROC Curves (AUROC) were calculated to determine the overall performance and optimal cut-offs of the sub-scales to predict depression, anxiety or both (common mental disorders, CMD) diagnosed by SCID. In this study, the criteria to select optimal cut-offs were (1) to maximize both Sensitivity (Se) and Specificity (Sp) and (2) to detect all potential cases by setting Se higher than Sp [21]. Data were analysed in STATA version 11 (StataCorp LP, College Station, Texas, United States of America, 2009).

### Ethics

The study was approved by the University of Melbourne’s Human Research Ethics Committee and the Viet Nam Medical Association’s Scientific Committee. Participants were given a small gift to the value of USD5 in recognition of their time away from income-generating work to contribute to the study. All participants were given an oral and written plain language description of the study and either signed a consent form, or those who could not write provided a thumbprint or verbal consent witnessed by an independent observer.

## Results

### Sample

Overall, 221/234 (94.4%) of potential participants agreed to join this study and provided complete data. Participants ranged in age from 20 to 44 years of age and were on average 27.7 years old (Table 2). Approximately one quarter of the women had completed high school (year 12) and most of them generated income through agricultural or manual work. Following the SCID interview, 26/221 (11.8%) women were diagnosed with depression; 24/221 (10.9%) with an anxiety disorder, 7/221 (3.2%) with co-morbid depression and an anxiety disorder. Overall, 43/221 (19.5%) met diagnostic criteria for at least one of the mental disorders.

**Table 2:** Sociodemographic characteristics of 221 Vietnamese women

	**Statistics**
*Age, mean years (SD)*	27.7 (5.5)
*Completed education, n (%)*	
Up to complete primary (years 1–5)	79 (35.7)
Complete secondary (years 6–9)	110 (49.8)
Complete high school (years 10–12)	19 (8.6)
Post-secondary	13 (5.9)
*Occupation, n (%)*	
Farmer	145 (65.6)
Factory, handcraft worker or retailer	51 (23.1)
Government or private officer	10 (4.5)
Not currently engaged in income-generating activity	15 (6.7)

SD: Standard deviation; n: number.

### Internal reliability

The internal consistency (Cronbach’s alpha) of each sub-scale was high (DASS21-D subscale 0.72; DASS21-A subscale 0.77; and DASS21-Ssubscale 0.70). The overall score, which includes all items, also had high consistency (Cronbach’s alpha = 0.88).

**Figure 1:**
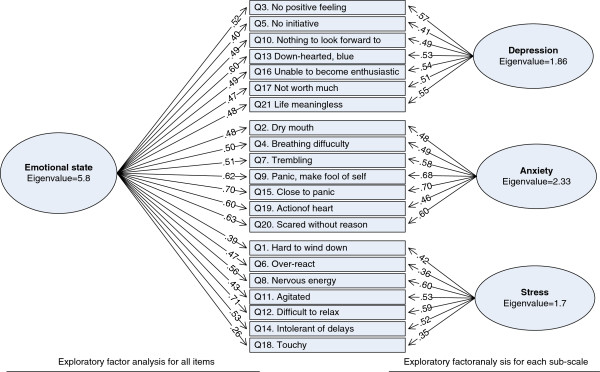
Exploratory factor analyses of the depression anxiety stress scale 21 items (DASS 21) in 221 Vietnamese women.

The results of the Exploratory Factor Analyses are presented in Figure 1. In the left-hand side, EFA was conducted for all 21 items. Only one factor (named emotional state) was found to be significant (eigenvalues > 1). All items loaded significantly on that factor except Q18 Touchy. The left-hand side of the Figure shows EFAs which were conducted for each sub-scale. For every sub-scale, only one factor was significant. All of the items of each sub-scale loaded significantly on the single factor.

Correlation coefficients between DASS21-D and DASS21-A was 0.65, DASS21-D and DASS21-S was 0.63, and DASS21-S and DASS21-A was 0.72.

### Validity and optimal cut-off points

The median scores and interquartile ranges of DASS21 sub–scales by diagnostic groups are presented in Table 3. The median scores of all sub-scales in women without depression or anxiety were significant lower than those in women diagnosed with depression or anxiety (p <0.05). However, the median scores between women diagnosed with depression or with anxiety were not significantly different in any sub-scale.

**Table 3:** Median scores and interquartile ranges of DASS21 sub–scales by diagnostic groups* in 221 Vietnamese women

	**DASS21- D**		**DASS21- A**		**DASS21- S**	
	**Median**	IQR***	**Median**	**IQR**	**Median**	**IQR**
All sample						
No mental health problem (N)	4	(0–8)	6	(2–10)	10	(6–14)
People with depression (D)	12	(10–18)	14	(10–22)	17	(10–24)
People with anxiety (A)	10	(6–18)	18	(10–26)	18	(14–25)
Tests of median differences**	D;A>N		D;A>N		D;A>N	

DASS21-D: Depression sub-scale of The Depression Anxiety and Stress Scales 21 items; DASS21-A: Anxiety sub-scale of The Depression Anxiety and Stress Scales 21 items; and DASS21-S: Stress sub-scale of The Depression Anxiety and Stress Scales 21 items.

Note: * Depression and anxiety were diagnosed by Structured Clinical Interviews for DSM IV; **nonparametric K-sample test on the equality of medians; ***IQR= interquartile range.

The summary of ROC analyses of each sub-scale, the combination of DASS21-D and DASS21-A, and the combination of three sub-scales to predict depression and/or anxiety are shown in Table 4. DASS21-D predicts depression better than anxiety with a higher cut-off, while DASS21-S predicts anxiety better than depression. However, DASS21-A predicts depression and anxiety with the same cut-off score. Combinations of two or three of the sub-scales strengthen the ability to predict depression and anxiety. Overall, none of the sub-scales or combinations could distinguish between depression and anxiety.

**Table 4:** ROC analyses of DASS21 to predict depression and/or anxiety* in Vietnamese women

	**Cut-off**	**Se (%)**	**Sp (%)**	**AURC (%)**
DASS21-D predicts				
Depression	>= 10	80.8	77.4	80.4
Anxiety	>= 8	70.8	69.0	76.1
CMD	>= 8	74.4	74.2	78.8
DASS21-A predicts				
Depression	>= 10	80.8	67.7	75.7
Anxiety	>= 10	79.2	67.0	80.6
CMD	>= 10	76.7	71.4	78.7
DASS21-S predicts				
Depression	>= 14	69.2	65.1	72.6
Anxiety	>= 14	79.2	66.0	80.6
CMD	>= 14	72.1	69.1	77.3
Combination of DASS21-**D** and DASS21-**A** predicts				
Depression	>= 20	80.7	71.3	80.2
Anxiety	>= 20	83.3	71.1	81.5
CMD	>= 20	79.1	75.8	81.7
Combination of DASS21-**D** and DASS21-**S** predicts				
Depression	>= 22	76.9	71.3	79.6
Anxiety	>= 22	87.5	72.1	82.2
CMD	>= 22	79.1	76.4	81.8
Combination of DASS21-**A** and DASS21-**S** predicts				
Depression	>= 24	69.2	69.2	75.7
Anxiety	>= 24	83.3	70.5	82.7
CMD	>= 24	74.4	74.2	79.0
Combination of three sub-scales predicts				
Depression	>= 36	80.8	75.4	79.2
Anxiety	>= 34	83.3	72.1	83.1
CMD	>= 34	79.1	77.0	81.9

DASS21-D: Depression sub-scale of The Depression Anxiety and Stress Scales 21 items; DASS21-A: Anxiety sub-scale of The Depression Anxiety and Stress Scales 21 items; and DASS21-S: Stress sub-scale of The Depression Anxiety and Stress Scales 21 items. CMD= common mental disorders included depression and anxiety.

Note: *Depression and anxiety were diagnosed by Structured Clinical Interviews for DSM IV.

## Discussion

This study used gold standard validation methods in a systematically recruited representative sample of women. It used standard analyses of internal reliability of psychometric instruments and means of establishing cut-off scores to detect clinically significant symptoms of depression and anxiety. We acknowledge the limitation that our sample was limited to women who had children of the same age. We used a sampling method which allowed us to recruit a representative sample of women with young children of Ha Nam, a typical rural northern province in Vietnam. The social-demographic characteristics of our sample are identical to that of rural women with young children in the North of Vietnam in The UNICEF Multiple Indicator Cluster Survey 2006 [22]. Therefore we believe that the data can be generalised with confidence to rural northern Vietnamese women with young children.

The results of EFA, analyses of the median scores of the three sub-scales by diagnostic groups and ROC analyses are consistent in indicating that in this setting, there is only one dimension of psychological functioning underlying the 21 items of the DASS21-Vietnam Validation (DASS21-V). None of the subscales was able to distinguish women experiencing depression from those with an anxiety disorder. These findings, are similar to our previous validations of other psychometric instruments in this setting [15], and suggest that while screening tools are able to detect clinically significant psychological states, they are not able to distinguish which state an individual woman is experiencing. It is possible that the symptoms which distinguish between depression and anxiety in Vietnamese women were absent in the screening tools and this warrants further investigation in future studies.

The other interpretation is that non-psychotic psychological morbidity is more accurately conceptualised in this setting as a continuum, rather than a distinct series of separate conditions. The term “common mental disorders” which refers to depression and anxiety is widely used in existing research to indicate that these are not readily distinguishable from each other in resource-constrained settings [1,2]. We have shown that in rural Vietnam there are the same risk factors for depressive and anxious states: intimate partner violence, low household wealth and coincidental adverse life events which are common among women in resource-constrained countries [17]. At community level therefore it might not be necessary to distinguish between these states as interventions such as universal psycho-educational programs or targeted supportive therapy to address these risks will be similar.

Consistent with previous studies [15,23], this study shows that psychological symptom screening tools have lower cut-offs in Vietnam compared with those in high-income settings. DASS21-D and DASS21-S which include only psychological items have optimal cut-offs in this study at the borderline of normal and mild levels of symptoms in the Australian classification (Table 1). DASS21-A in which three of the seven items are somatic symptoms has a cut-off score to detect anxiety in this study falling in the range of the moderate level in the Australian classification. The reason may be that women with depression and anxiety tend to endorse more somatic symptoms than negative emotions.

These data demonstrate that both the combination of DASS21-D subscale and DASS21-A subscale and the combination of all three sub-scales generate a sensitive tool to screen for common mental disorders among women with young children in this setting. We suggested the combination of 21 items of DASS21 for primary health care because it has higher sensitivity and specificity than either any of the single sub-scales or combinations of two sub-scales. The optimal cut-off score of this tool is equal to or more than 34 which yields optimal sensitivity (79.1%) and specificity (77.0%) to detect common mental disorders including depression and anxiety. Using the full scale with 42 items may have different results. However, it would increase the burden and there would be no difference in treatment.

## Conclusions

In summary, our data indicate that DASS21-V as a single scale has good internal reliability and is sensitive to detect common mental disorders in rural northern Vietnamese women with young children. Using the sub-scales to distinguish between depression and anxiety is not however appropriate for this setting. Overall, the DASS 21-V is a comprehensible and psychometrically sound tool which can be used in primary health care, community interventions, or as a screening tool at clinical settings in rural Viet Nam.

## Competing interests

The authors declare that they have no conflicts of interest.

## Authors’ contributions

All authors contributed to designing and conducting the study and analysis and interpretation of data. TDT and JF wrote the first draft of the manuscript and all authors reviewed it and have approved the final manuscript.

## Pre-publication history

The pre-publication history for this paper can be accessed here:

http://www.biomedcentral.com/1471-244X/13/24/prepub
